# Silica coating influences the corona and biokinetics of cerium oxide nanoparticles

**DOI:** 10.1186/s12989-015-0106-4

**Published:** 2015-10-12

**Authors:** Nagarjun V. Konduru, Renato J. Jimenez, Archana Swami, Sherri Friend, Vincent Castranova, Philip Demokritou, Joseph D. Brain, Ramon M. Molina

**Affiliations:** Molecular and Integrative Physiological Sciences Program, Department of Environmental Health, Harvard T.H. Chan School of Public Health, 665 Huntington Avenue, Boston, MA 02115 USA; National Institute for Occupational Safety and Health, Morgantown, WV USA; Department of Basic Pharmaceutical Sciences, School of Pharmacy, West Virginia University, P.O. Box 9530, Morgantown, WV 26506 USA

**Keywords:** Nanoceria, Bioavailability, Protein corona, Silica

## Abstract

**Background:**

The physicochemical properties of nanoparticles (NPs) influence their biological outcomes.

**Methods:**

We assessed the effects of an amorphous silica coating on the pharmacokinetics and pulmonary effects of CeO_2_ NPs following intratracheal (IT) instillation, gavage and intravenous injection in rats. Uncoated and silica-coated CeO_2_ NPs were generated by flame spray pyrolysis and later neutron-activated. These radioactive NPs were IT-instilled, gavaged, or intravenously (IV) injected in rats. Animals were analyzed over 28 days post-IT, 7 days post-gavage and 2 days post-injection.

**Results:**

Our data indicate that silica coating caused more but transient lung inflammation compared to uncoated CeO_2_. The transient inflammation of silica-coated CeO_2_ was accompanied by its enhanced clearance. Then, from 7 to 28 days, clearance was similar although significantly more ^141^Ce from silica-coated (35 %) was cleared than from uncoated (19 %) ^141^CeO_2_ in 28 days. The protein coronas of the two NPs were significantly different when they were incubated with alveolar lining fluid. Despite more rapid clearance from the lungs, the extrapulmonary ^141^Ce from silica-coated ^141^CeO_2_ was still minimal (<1 %) although lower than from uncoated ^141^CeO_2_ NPs. Post-gavage, nearly 100 % of both NPs were excreted in the feces consistent with very low gut absorption. Both IV-injected ^141^CeO_2_ NP types were primarily retained in the liver and spleen. The silica coating significantly altered the plasma protein corona composition and enhanced retention of ^141^Ce in other organs except the liver.

**Conclusion:**

We conclude that silica coating of nanoceria alters the biodistribution of cerium likely due to modifications in protein corona formation after IT and IV administration.

## Background

With rapid growth in nanotechnology-enabled consumer products, engineered nanomaterials (ENMs) are increasingly common. At the same time, there are rising public concerns about adverse effects of ENMs on human health and the environment. Among the ENMs introduced into the global nanotechnology market, nanoceria (CeO_2_) has moved to the fore with a wide array of applications. The ability of cerium to switch oxidation states between Ce (III) and Ce (IV) is crucial for many nanobiomedical applications [1–4]. Further, parameters such as the method employed to synthesize CeO_2_, its particle size, and the extent of doping with other agents may alter the cerium oxidation state [3, 5].

The toxicity data on CeO_2_ from studies undertaken during the last decade are mixed and report a range of biological effects [6]. A number of *in vivo* and *in vitro* studies evaluating the biological effects of CeO_2_ have reported toxicity and oxidative stress [7–10]. However, recently there are also reports highlighting putative antioxidant activity of CeO_2_ and its ability to protect against oxidative stress-driven disorders [11–13]. Baer *et al.* have shed light on the influence of synthesis method, particle size and aging of CeO_2_ on biological outcomes [5]. Many studies have underscored the need for defining nanoparticle characteristics employed in biological studies. There are conflicting data on CeO_2_ toxicity and the consequences of different concentrations [14, 15]. It is important to consider the extent of particle agglomeration in air and liquid media as a crucial factor contributing to discrepancies between *in vivo* inhalation versus instillation studies. Nanoparticle agglomeration is primarily influenced by NP intrinsic properties such as surface chemistry, charge, and primary particle size, but also from properties of suspending medium such as ionic strength [16–19].

Nanoparticle recognition by alveolar macrophages is a determinant of effective lung clearance. There is evidence that particle agglomeration aids in promotion of effective phagocytosis in alveolar macrophages; smaller (<100 nm) and more abundant structures may make macrophage mediated “surveillance” less effective [20]. Scientists are creating nanoparticles with functional surfaces designed to reduce inflammogenicity and lower toxicity while improving useful physicochemical properties. Developing strategies to mitigate toxicity of NPs without altering their core properties (a safer-by-design approach) is a vigorously pursued area of research [21–23]. In some cases, surface encapsulation of nanomaterials with a thin layer of amorphous silica renders them less cytotoxic and reduces DNA damage. Coating nanoparticles with amorphous silica can enhance nanoparticle stability in colloidal suspensions and facilitate effective uptake by professional phagocytes, stem cells, and other cell types with reduced toxicity [24–26]. Unlike crystalline silica that induces sustained inflammation and resultant fibrosis, amorphous silica evokes a transient and reversible inflammatory response [27].

We recently investigated the pulmonary clearance and extrapulmonary translocation of radiolabled Ce after intratracheal instillation of CeO_2_ [15]. Our study showed that only 12 % of the instilled Ce dose was cleared from the rat lung during 28 days post-exposure. In another investigation, we found that inhalation of CeO_2_ caused more lung injury and inflammation than CeO_2_ coated with amorphous silica after one day post-exposure [28]. Previous reports have proposed that the protein corona formed on particles can influence biological effects [29]. To our knowledge this is the first study investigating the influence of surface properties of cerium oxide nanoparticles on protein corona formation, pulmonary effects, and the translocation and distribution of nanoceria after pulmonary and intravenous administration. We employed amorphous silica coating as a model to test the hypothesis that surface coating of CeO_2_ would alter its protein corona and thus influence the biokinetics of the core nanoceria. We chose nanoceria due to its slow lung clearance and relatively low solubility [15, 30–32]. The aim of our study was to compare the clearance kinetics and bioavailability of cerium after intratracheal, intragastric, and intravenous administration of silica-coated versus uncoated CeO_2_ in rats.

## Results

### Synthesis and characterization of CeO_2_ and silica-coated CeO_2_

**Fig. 1:**
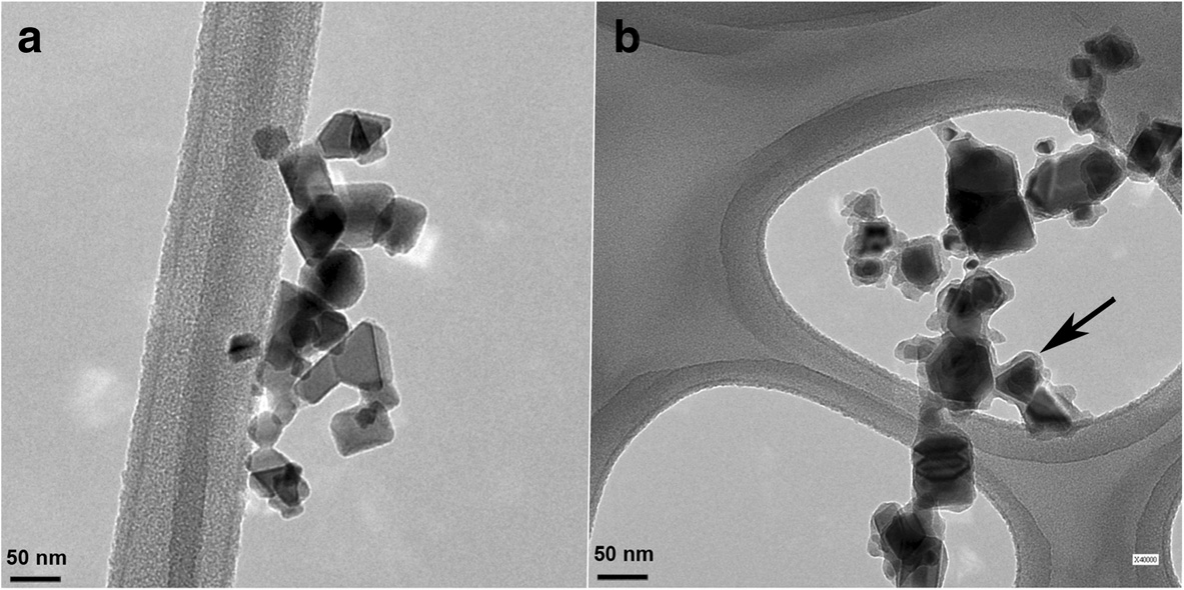
Appearance of CeO_2_ NPs used in this study. **a** Electron micrograph of uncoated and **b** silica-coated CeO_2_ NPs. Arrow shows a thin silica coating

Uncoated and silica-coated CeO_2_ were made by flame spray pyrolysis using the Versatile Engineered Nanomaterial Generation System (VENGES) at Harvard University [33]. Detailed physicochemical and morphological characterization of these NPs was reported earlier [21, 28]. In summary, the uncoated and silica-coated CeO_2_ had a cubic fluorite-like structure (Fig. 1). A nanothin (2–4 nm) amorphous silica layer hermetically encapsulated the CeO_2_ core in a coating reactor after their initial synthesis in an aerosol reactor [21] (Fig. 1b). The silica coating on the surface was revealed as fine optically transparent film surrounding the dark and opaque CeO_2_, as verified by X-ray diffraction (XRD) and electron microscopy analyses. The average crystal size of the primary uncoated and silica-coated NPs was 32.9 and 32.6 nm, respectively. Their specific surface areas (SSA) were 28 m^2^/g (uncoated) and 27.8 m^2^/g (silica-coated) (Table 1). The extent of the silica coating was assessed by X-ray photoelectron spectroscopy and by photocatalytic methods. The persistence of the silica coating in the lungs of rats was at least 3 days after inhalation [34].

Assessments by dynamic light scattering (DLS) showed that as an aqueous dispersion the particles essentially behaved as “nanoagglomerates” of 136 ± 1.1 nm (uncoated) and 208 ± 2.9 nm (silica-coated). The hydrodynamic diameters of the two CeO_2_ types are shown in Table 1. The zeta potential of NP suspensions was also evaluated in distilled water. Uncoated CeO_2_ exhibited a positive zeta potential (34.5 ± 3.1 mV) and the silica coating changed the zeta potential to negative −26.8 ± 0.3 mV (Table 1). DLS analysis was also performed on both nanoceria after *in vitro* incubation with harvested rat bronchoalveolar lining (BAL) fluid to determine if the lipoprotein corona alters agglomeration size and zeta potential. We found that this corona significantly increased the hydrodynamic diameter (136 to 1463 nm) and changed the zeta potential (34.5 to −20.8 mV) of uncoated CeO_2_. The effects of the lipoprotein corona on silica-coated CeO_2_ were more modest (Table 1). After incubation in rat plasma and the formation of the protein corona, the hydrodynamic diameters of both CeO_2_ NP types were significantly increased and the surface charge of uncoated CeO_2_ was also altered from positive to negative zeta potential (Table 1). Similar to protein corona formed with BAL incubation, the increase in D_H_ with plasma protein corona formation was more pronounced with uncoated CeO_2_ NPs.

**Table 1:** Physicochemical characterization of nanoparticles used

	CeO_2_ in DI water	Silica-coated CeO_2_ in DI water	CeO_2_ in BAL	Silica-coated CeO_2_ in BAL	CeO_2_ in plasma	Silica-coated CeO_2_ in plasma
SSA (m^2^/g)	28.0	27.8	N.A.	N.A.	N.A.	N.A.
D_xrd_ (nm)	32.9	32.6	N.A.	N.A.	N.A.	N.A.
D_H_ (nm)	136 ± 1	208 ± 3	1463 ± 88	460 ± 12	2572 ± 372	242 ± 3
ζ(mv)	34.5 ± 3.1	−26.8 ± 0.3	−20.8 ± 3.4	−15.4 ± 2.0	−25.2 ± 2.8	−31.8 ± 2.7

*SSA* - specific surface area

*D*_*xrd*_ - primary particle size based on X-ray diffraction

*D*_*H*_ - hydrodynamic diameter

*ζ* - zeta potential

*N.A.* - not applicable

### Pulmonary responses to intratracheally instilled CeO_2_ and silica-coated CeO_2_

**Fig. 2:**
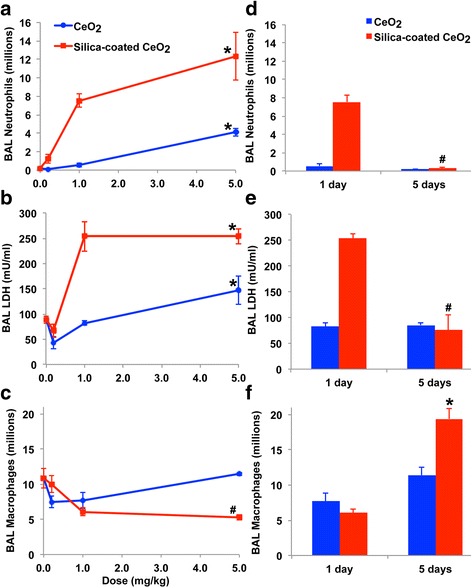
Bronchoalveolar lavage analysis after IT instillation of uncoated or silica-coated CeO_2_ NPs. **a** Dose-dependent increases in lavaged neutrophils and **b** lactate dehydrogenase levels in BAL at 24 h post-instillation. **c** Lavaged macrophages increased with uncoated but decreased with silica-coated CeO_2_ at the highest NP dose. **d** Lavaged neutrophils and **e** lactate dehydrogenase, MPO and albumin (data not shown) returned to normal levels but **f** macrophage recruitment was observed at 5 days post-instillation of 1 mg/kg CeO_2_. (* increased, # decreased, *P* < 0.05, MANOVA. Data are mean ± SEM, *n* = 5/group)

We compared the pulmonary responses of rats to uncoated versus silica-coated CeO_2_ at 1 and 5 days after IT instillation in rats as described previously [35]. This experiment was performed to also determine the safe dose for intratracheal instillation of CeO_2_ and silica-coated CeO_2_ NPs where inflammation or injury was minimal. Groups of 6 rats (272 ± 13 g body weight) were instilled with 0.2, 1 or 5 mg/kg of each type of CeO_2_. Control animals were instilled with an equivalent volume of distilled water. We found that coated and uncoated CeO_2_ NPs induced a dose-dependent injury and inflammation as indicated by increased neutrophils (Fig. 2a) in the BAL fluid at 24 h post-instillation. Both NPs also increased the levels of myeloperoxidase (MPO), albumin and lactate dehydrogenase (LDH) (Fig. 2b). Interestingly, the numbers of lavaged macrophages increased for uncoated and decreased for silica-coated CeO_2_ with increasing dose (Fig. 2c). At 0.2 and 1 mg/kg doses, only the silica-coated CeO_2_ instilled rats showed elevated LDH, MPO, and albumin levels. However, five days post-dosing with 1 mg/kg of silica-coated CeO_2_ there were decreased PMN counts (Fig. 2d). At this time, there were also reductions in other inflammatory biomarkers such as MPO, albumin and LDH (Fig. 2e). However, significant increase in macrophage numbers was observed in silica-coated CeO_2_ groups (Fig. 2f).

### In vivo clearance and translocation of ^141^CeO_2_ and silica-coated ^141^CeO_2_ after IT instillation in rats

**Fig. 3:**
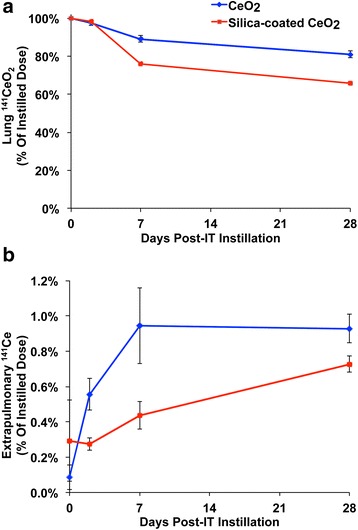
Biokinetics of IT-instilled ^141^CeO_2_ nanoparticles. **a** Lung clearance of both CeO_2_ NPs was slow. Although similar during the first two days post-IT instillation, it was different between from day 2 to day 7. Approximately 22 % of silica-coated CeO_2_ and only ~8 % of uncoated CeO_2_ total dose cleared the lungs during this period. By 28 days post-instillation, 81 % of uncoated and 66 % of silica-coated CeO_2_ remained in the lungs. **b** Translocated ^141^Ce from the lungs gradually accumulated in extrapulmonary organs. By 28 days, only 0.9 % of instilled ^141^Ce dose from uncoated and 0.7 % from silica-coated CeO_2_ were retained in all extrapulmonary organs examined. Data are mean ± SEM, *n* = 5/group

The lung levels of ^141^Ce after a single IT instillation of either radioactive uncoated CeO_2_ or silica-coated CeO_2_ were evaluated in rats for 28 days. Animals were sacrificed at 5 min, and 2, 7 and 28 days post-instillation and various organs were collected to determine the retained cerium concentration. The lung clearance profiles for both nanoparticle types showed no differences during the first two days post-IT instillation. Interestingly, the lung clearance was markedly different between day 2 and day 7 for the two NPs (Fig. 3a). We observed that ~22 % of the ^141^Ce from the silica-coated CeO_2_ and only ~8 % of the ^141^Ce from the uncoated CeO_2_ dose disappeared from the lungs during this period. Between day 7 and day 28 post-IT instillation, the difference in the fraction of cleared NPs was statistically significant but relatively small (8.1 % for uncoated ^141^CeO_2_ vs. 10.4 % for silica-coated CeO_2_). By 28 days post-instillation, ~81 % of uncoated CeO_2_ still remained in the lungs. Coating of CeO_2_ with amorphous silica enhanced the overall clearance of CeO_2_ by an additional 16 %.

**Fig. 4:**
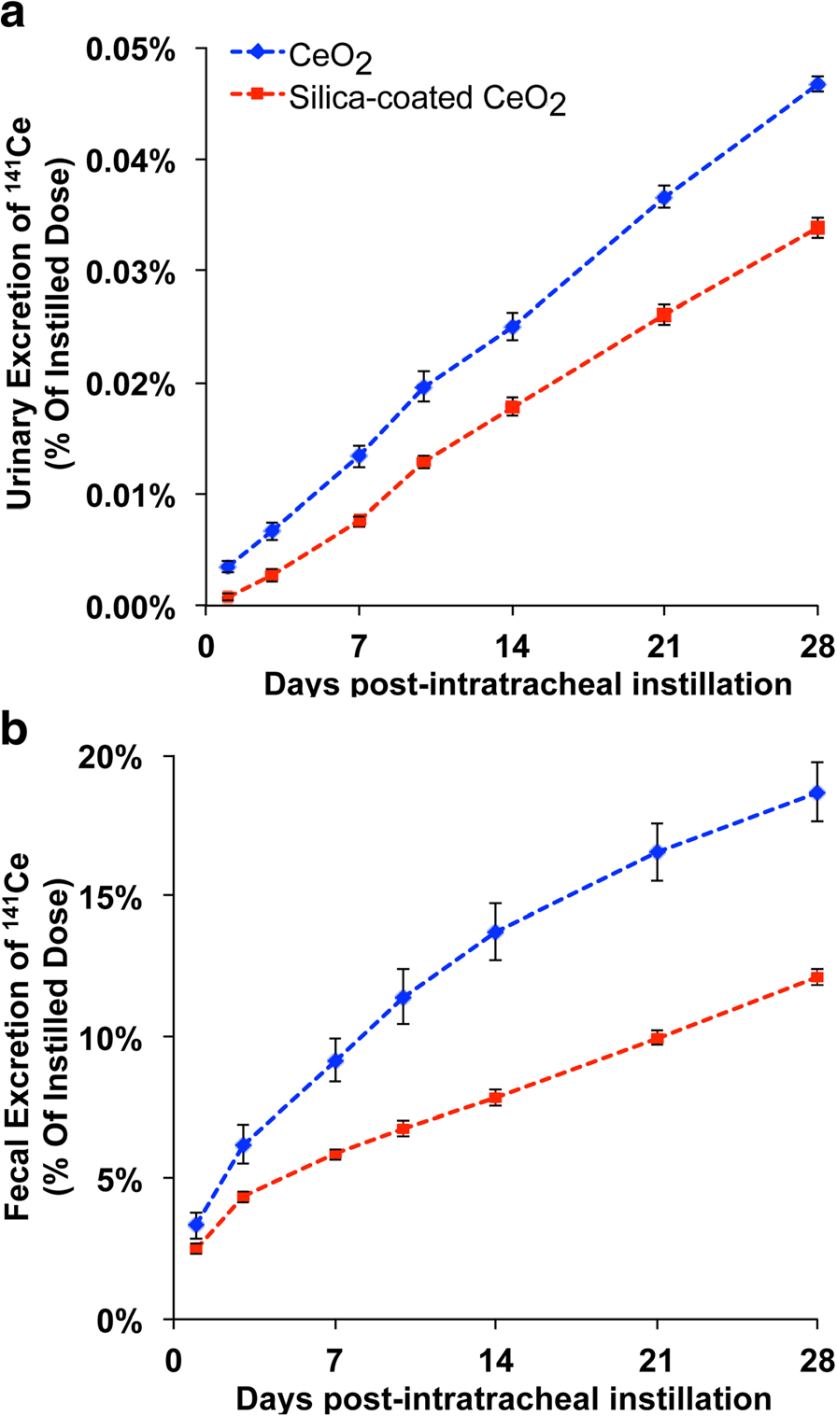
Elimination of ^141^Ce post-IT instillation. **a**. Only 0.03 % – 0.05 % was excreted in the urine in 28 days. **b** However, 19 % of ^141^Ce from uncoated and 12 % from silica-coated CeO_2_ was excreted in the feces. Data are mean ± SEM, *n* = 5/group

Translocation of radioactive cerium from the lungs to other organs was evaluated by measuring ^141^Ce in the different collected tissues. Low detectable fractions of radioactivity for Ce from both NP types were found in the liver, bone/bone marrow, spleen and kidneys (<1 %) (Fig. 3b). Estimated tissue cerium concentration in these organs were higher for uncoated CeO_2_ (Table 2). The elimination of ^141^Ce from both particle types was mostly via the feces (Fig. 4b) and to a much lesser extent via the urine (Fig. 4a). Furthermore, we found that the total recovered ^141^Ce in examined tissues, feces, and urine was significantly higher in uncoated than silica-coated CeO_2_ (Figs. 3 and 4). In the case of silica-coated CeO_2_, we speculate that the missing radioactivity may have been in organs not examined such as lymph nodes, adipose tissue, pancreas, adrenals, teeth, nails, tendons, nasal tissues, and the rest of the head.

**Table 2:** Cerium concentration at 28 days post-instillation of uncoated or silica-coated CeO_2_ NPs

	CeO_2_	Silica-coated CeO_2_
	ng/g ± SE	ng/g ± SE
Lungs	136836.67 ± 4084.19	95193.25 ± 1766.39 *
Liver	59.35 ± 10.19	33.10 ± 2.78 *
Bone	38.13 ± 5.14	21.97 ± 2.87 *
Cecum	33.62 ± 10.95	24.15 ± 6.58
Large intestine	28.11 ± 8.94	24.33 ± 8.01
Bone marrow	16.55 ± 2.55	9.44 ± 1.05 *
Spleen	13.90 ± 6.21	3.83 ± 0.59
Stomach	12.77 ± 2.09	16.82 ± 8.64
Kidneys	10.40 ± 1.13	5.52 ± 0.35 *
Small intestine	10.14 ± 1.49	7.94 ± 2.20
Heart	1.95 ± 0.54	0.21 ± 0.08 *
Testes	0.54 ± 0.08	0.13 ± 0.04 *
Skeletal muscle	0.46 ± 0.21	0.20 ± 0.09
Brain	0.43 ± 0.24	0.15 ± 0.11
Skin	0.38 ± 0.08	0.16 ± 0.01 *
Plasma	0.14 ± 0.09	0.04 ± 0.04
RBC	0.04 ± 0.04	0.08 ± 0.06

Data are mean ± SE ng/g cerium concentration, *n* = 5/group

Ce concentration was estimated (ng/μCi_NPs_ x μCi/g_tissue_)

*P < 0.05, CeO_2_ vs. silica-coated CeO_2_

### Biodistribution of CeO_2_ within the lungs and protein corona formation

**Fig. 5:**
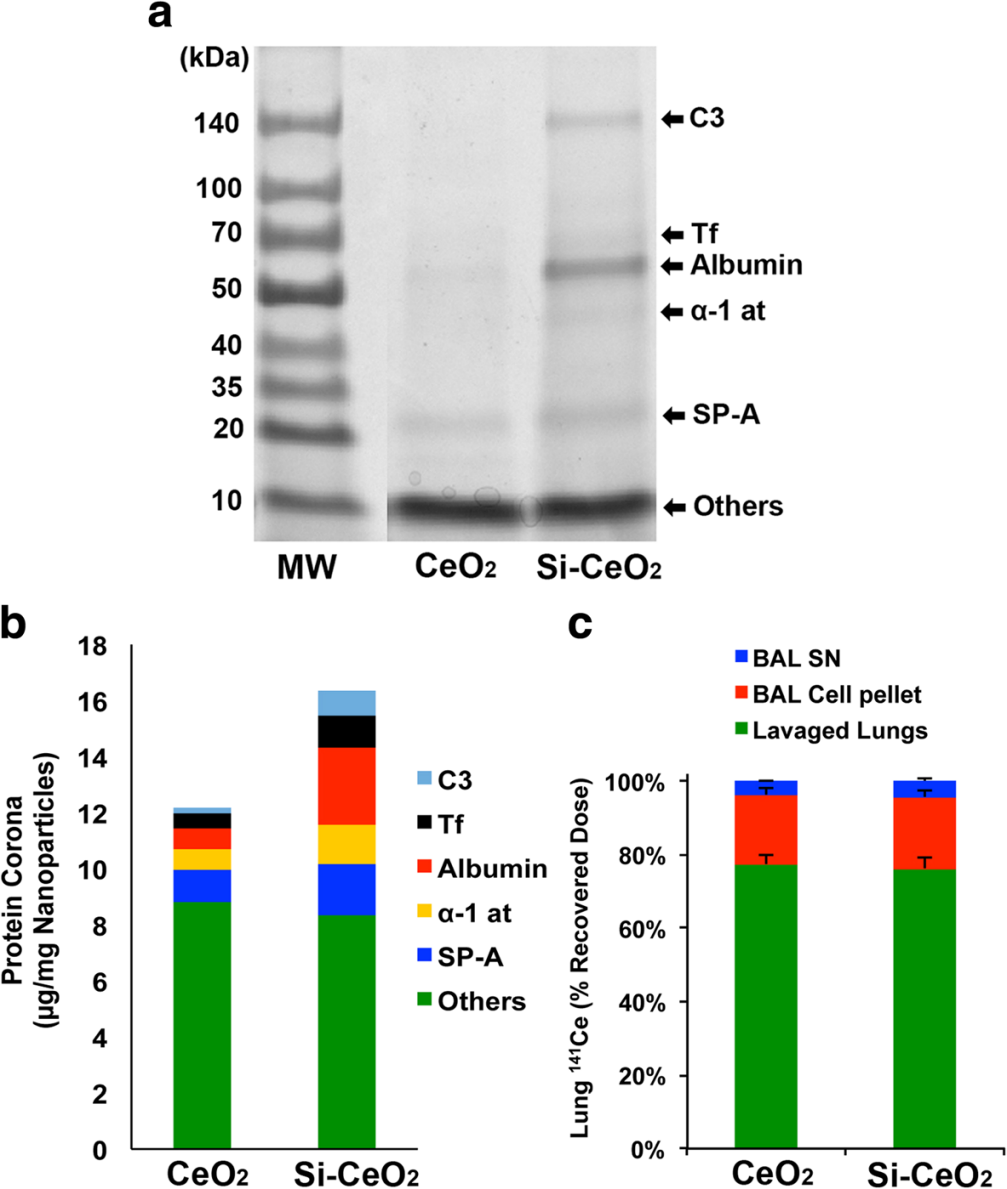
Analysis of nanoparticles after incubation with BAL fluid. **a** NP-bound rat BAL proteins were analyzed by 1D gel electrophoresis and Mass Spectrometry. The molecular weights (kDa) of reference proteins are shown in lane MW. Five proteins identified by LC-MS are indicated on right. **b** LC-MS profiles of the same five proteins show the influence of silica coating on the protein corona profile. **c** Compartmental distribution of neutron activated uncoated and silica-coated CeO_2_ at 24 h post-instillation. No significant differences in distribution were observed between the two CeO_2_ NPs. Data are mean ± SEM, *n* = 5/group

**Fig. 6:**
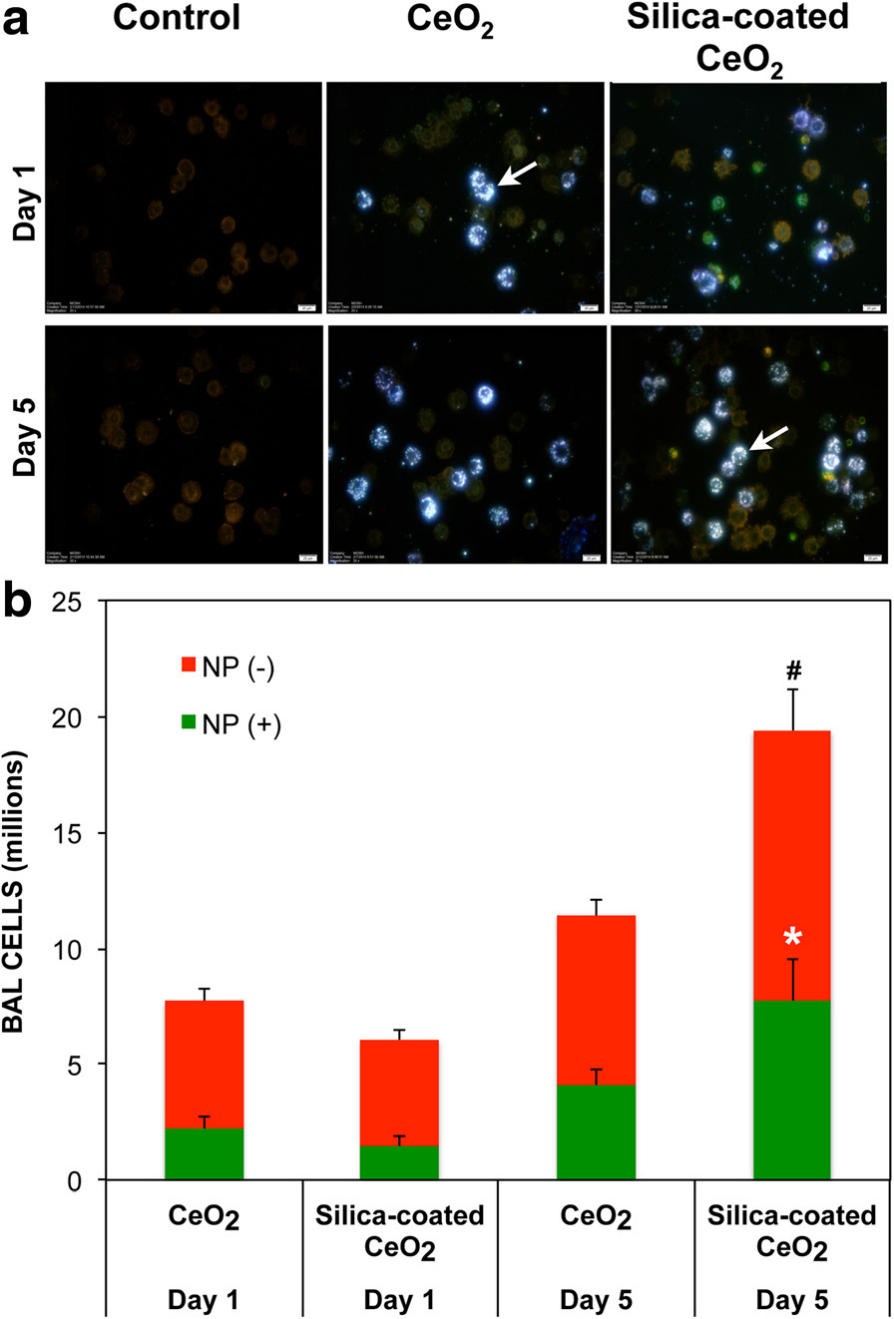
Quantitative assessment of uptake of CeO_2_ by alveolar macrophages at 24 h post-instillation. BAL cells were analyzed using hyperspectral imaging. **a** The image shows uncoated and silica-coated CeO_2_ mapped as bright pixels (pointed arrows) inside the cells. BAL cells isolated at 24 h and 5 days after IT-instillation were scored. **b** Numbers of macrophages with or without internalized CeO_2_ at 1 and 5 days post-instillation. Significantly more cells with ingested silica-coated CeO_2_ were seen at 5 days. Data are mean ± SEM, *n* = 3 rats/group, *n* = 3000 cells scored/group. * *P* < 0.05, Student *t* test

Since the protein corona on NP surfaces may modulate their cell interaction and overall biological effects, we examined the composition of adsorbed proteins on the NP surface when incubated with collected cell-free BAL fluid. First, we found that incubation of NPs in concentrated BAL fluid significantly altered their aggregate sizes (Table 1). Compared with the suspension in deionized water, both nanoceria types exhibited larger and more variable hydrodynamic diameter. Uncoated nanoceria also formed larger agglomerates than the coated NPs. In addition, we found that the total amount of adsorbed protein was significantly higher in silica-coated than uncoated CeO_2_ especially albumin, C3, and transferrin (Fig. 5a, b). However, we found no differences in the quantitative distribution of the two NP types 24 h post-IT instillation among the three measured compartments (Fig. 5c). The majority of ^141^Ce activity was associated with the lavaged lungs. Additionally, hyperspectral imaging analysis, to determine the extent of NP uptake in BAL cells after 5 days post-IT instillation, revealed a higher number of particle-containing cells in the silica-coated than uncoated group (Fig. 6).

### Biodistribution of uncoated and silica-coated CeO_2_ after gavage administration in rats

**Fig. 7:**
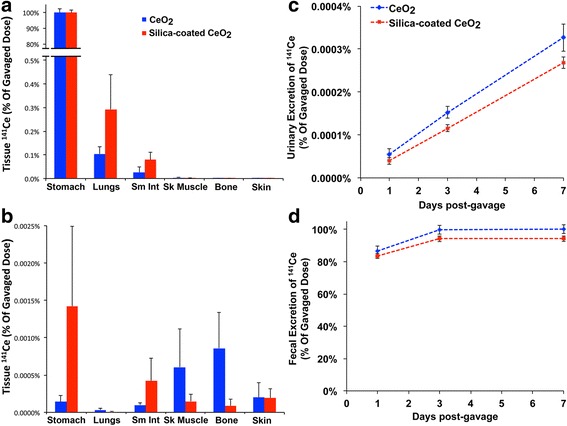
Tissue distribution of ^141^Ce post-gavage. **a** Immediately post-gavage, nearly 100 % of both CeO_2_ were recovered in the stomach and to much lesser extent in other organs. **b** At 7 days post-gavage, the total tissue ^141^Ce detected in all organs examined was negligible (0.003 ± 0.001 %). **c** By 7 days post-gavage, less than 0.0004 % of dose was excreted in the urine. **d** Elimination of ^141^Ce via the feces was nearly 100 % from uncoated and 94 % from silica-coated CeO_2_. Data are mean ± SEM, *n* = 5/group

At 5 min and 7 days post-gavage of uncoated CeO_2_ or silica-coated CeO_2_ we measured absorption of ^141^Ce from the gut. As expected, nearly 100 % of the dose was recovered at 5 min in the stomach for both types of NPs (Fig. 7a). The ^141^Ce levels in tissues other than the gastrointestinal (GI) tract were extremely low (0.004 % for uncoated, 0.002 % for silica-coated CeO_2_) by day 7 (Fig. 7b). Very low levels of ^141^Ce were excreted in the urine (Fig. 7c) and nearly 99 % of both CeO_2_ NPs was excreted in feces by day 7 (Fig. 7d). As there was very low radioactivity detected in any of the collected organs and in urine samples over a period of 7 days, we conclude that both uncoated and silica-coated CeO_2_ do not significantly translocate through the intestinal barrier. Tissue concentration of cerium at 7 days post-gavage is shown in Table 3.

**Table 3:** Cerium concentration in different tissues at 7 days after gavage administration of uncoated or silica-coated CeO_2_ NPs

	CeO_2_	Silica-coated CeO_2_
	ng/g ± SE	ng/g ± SE
Lungs	0.27 ± 0.16	0.07 ± 0.03
Liver	0.11 ± 0.04	0.07 ± 0.03
Bone	0.55 ± 0.31	0.06 ± 0.06
Cecum	0.22 ± 0.08	0.18 ± 0.13
Large intestine	0.76 ± 0.72	0.20 ± 0.15
Bone marrow	0.00 ± 0.00	0.25 ± 0.25
Spleen	0.06 ± 0.06	0.15 ± 0.15
Stomach	0.31 ± 0.18	2.64 ± 1.84
Kidneys	0.13 ± 0.13	0.09 ± 0.04
Small intestine	0.12 ± 0.05	0.39 ± 0.27
Heart	0.34 ± 0.21	0.35 ± 0.21
Testes	0.04 ± 0.04	0.09 ± 0.04
Skeletal muscle	0.06 ± 0.05	0.01 ± 0.01
Brain	0.05 ± 0.05	0.00 ± 0.00
Skin	0.04 ± 0.04	0.04 ± 0.02
Plasma	0.04 ± 0.04	0.03 ± 0.03
RBC	0.00 ± 0.00	0.03 ± 0.03

Data are mean ± SE ng/g cerium concentration, *n* = 5/group

Ce concentration was estimated (ng/μCi_NPs_ x μCi/g_tissue_)

No significant difference was observed between the two group

### Tissue distribution of ^141^CeO_2_ and silica-coated ^141^CeO_2_ NPs after intravenous injection

**Fig. 8:**
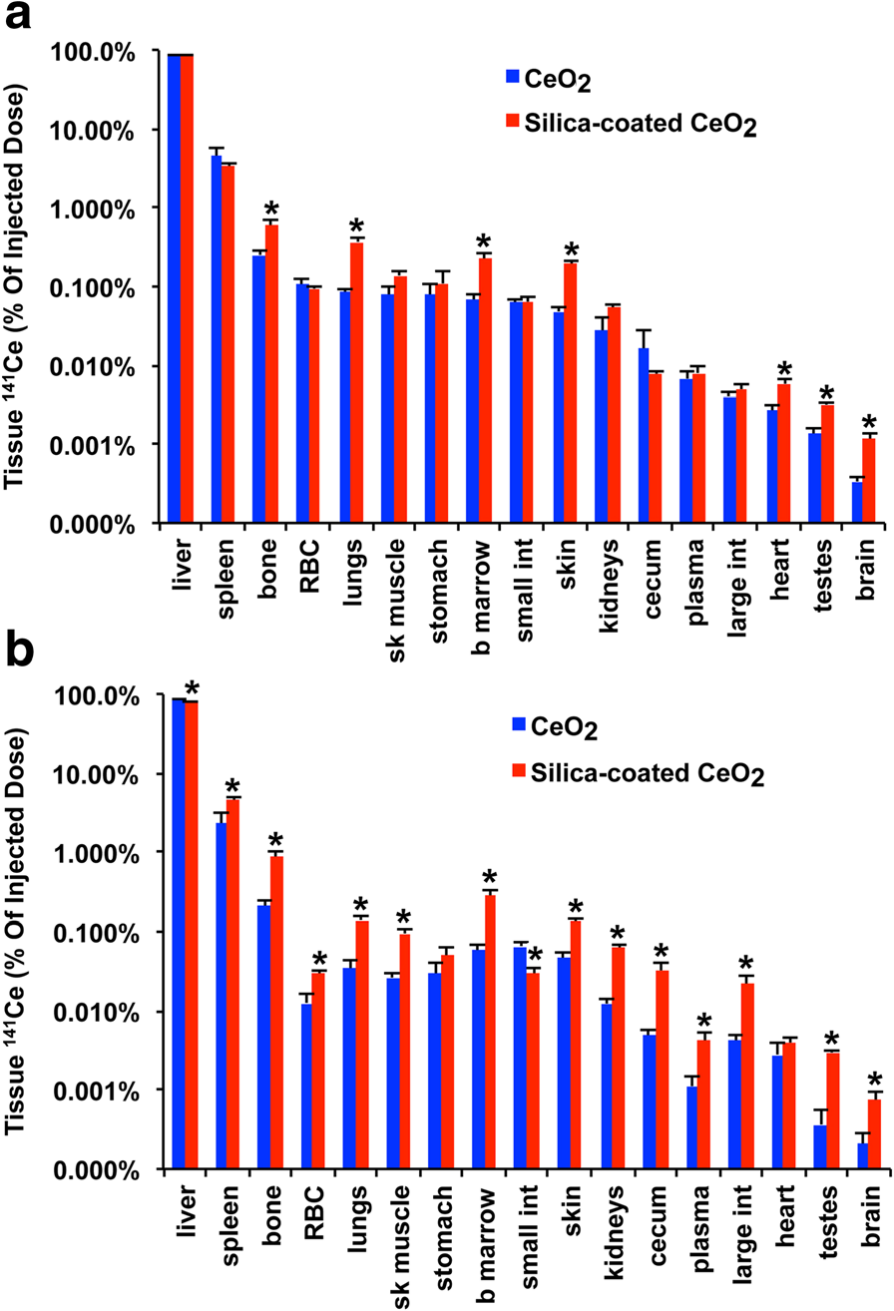
Tissue distribution of ^141^Ce post-IV injection of CeO_2_ NPs. **a** At 2 h post-injection, 87 % of ^141^Ce dose was recovered in the liver, and lower percentages in blood, spleen, bone, and bone marrow from both CeO_2_ group. **b** Over a period of 2 days, ^141^Ce levels in the liver decreased from 87 % to 80 % in the silica-coated group with accompanying increases in the spleen, bone and bone marrow. * *P* <0.05, MANOVA. Data are mean ± SEM, *n* = 5/group

The distribution of intravenously injected NPs at 2 h and 2 days post-injection is shown in Fig. 8a and b, respectively. Radioactive ^141^Ce from both NP types was predominantly retained in the liver, spleen, and bone, organs that typically take up circulating particles by macrophages with access to the blood. The silica coating led to a redistribution of ^141^Ce over a period of 2 days from the liver to the spleen and other organs (Fig. 8b). The silica coating also enhanced the tissue concentration of ^141^Ce in several organs but decreased in the liver (Tables 4 and 5).

**Table 4:** Cerium concentration in different tissues at 2 hours after intravenous injection of uncoated or silica-coated CeO_2_ NPs

	CeO_2_	Silica-coated CeO_2_
	ng/g ± SE	ng/g ± SE
Lungs	14.37 ± 1.79	54.62 ± 4.93 *
Liver	1951.79 ± 44.49	1663.68 ± 67.67 *
Bone	3.38 ± 0.44	6.42 ± 1.14 *
Cecum	0.62 ± 0.43	0.21 ± 0.03
Large intestine	0.36 ± 0.09	0.28 ± 0.05
Bone marrow	1.73 ± 0.34	4.67 ± 0.70 *
Spleen	1680.71 ± 357.91	1204.38 ± 171.76
Stomach	3.77 ± 1.67	4.77 ± 2.38
Kidneys	3.09 ± 1.44	5.27 ± 0.29
Small intestine	1.37 ± 0.20	1.12 ± 0.14
Heart	0.77 ± 0.15	1.41 ± 0.26
Testes	0.09 ± 0.02	0.19 ± 0.01 *
Skeletal muscle	0.16 ± 0.05	0.23 ± 0.01
Brain	0.04 ± 0.00	0.11 ± 0.02 *
Skin	0.20 ± 0.03	0.68 ± 0.04 *
Plasma	0.14 ± 0.03	0.14 ± 0.03
RBC	2.66 ± 0.51	2.07 ± 0.23

Data are mean ± SE ng/g cerium concentration, *n* = 5/group

Ce concentration was estimated (ng/μCi_NPs_ x μCi/g_tissue_)

*P < 0.05, CeO_2_ vs. silica-coated CeO_2_

**Table 5:** Cerium concentration in different tissues at 2 days after intravenous injection of uncoated or silica-coated CeO_2_ NPs

	CeO_2_	Silica-coated CeO_2_
	ng/g ± SE	ng/g ± SE
Lungs	5.65 ±1.53	23.15 ± 2.83 *
Liver	1904.43 ± 93.22	1652.57 ± 38.07 *
Bone	2.72 ± 0.64	10.95 ± 1.61 *
Cecum	0.15 ± 0.02	0.95 ± 0.26 *
Large intestine	0.21 ± 0.03	1.07 ± 0.29 *
Bone marrow	1.42 ± 0.28	6.64 ± 1.23 *
Spleen	1096.02 ± 333.49	1822.33 ± 181.83
Stomach	2.01 ± 0.89	3.29 ± 1.25
Kidneys	1.32 ± 0.18	6.32 ± 0.58 *
Small intestine	1.47 ± 0.14	0.63 ± 0.12 *
Heart	0.75 ± 0.26	1.05 ± 0.12
Testes	0.02 ± 0.01	0.20 ± 0.02 *
Skeletal muscle	0.05 ± 0.01	0.18 ± 0.02 *
Brain	0.02 ± 0.01	0.08 ± 0.02 *
Skin	0.19 ± 0.03	0.52 ± 0.08 *
Plasma	0.02 ± 0.01	0.09 ± 0.02 *
RBC	0.30 ± 0.69	0.69 ± 0.03 *

Data are mean ± SE ng/g cerium concentration, *n* = 5/group

Ce concentration was estimated (ng/μCi_NPs_ x μCi/g_tissue_)

*P < 0.05, CeO_2_ vs. silica-coated CeO_2_

**Fig. 9:**
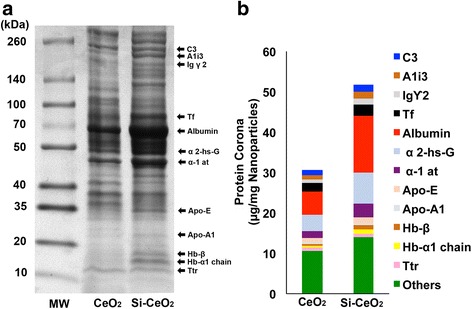
Analysis of nanoparticle protein corona after incubation in plasma. **a** Analysis of NP-bound rat plasma proteins by 1D gel electrophoresis. The molecular weights (kDa) of reference proteins are shown in lane MW. Twelve proteins identified by LC-MS are indicated on right. **b** LC-MS profiles of the same twelve proteins and influence of silica coating on the corona profile

To determine the influence of silica coating on NP-plasma protein interactions, we analyzed the hydrodynamic diameters of NPs and characterized the protein corona formed after incubation of NPs in rat plasma *in vitro*. We found significant increases in agglomerate sizes of both NP types compared to when suspended in protein-free deionized water (Table 1). We also found differences in the protein corona composition between the 2 NP types (Fig. 9a, b). The fecal excretion of ^141^Ce post-injection of NPs during the first 24 h was far lower than after IT instillation (0.05 % v. 3 %), suggesting that some CeO_2_ NPs in the lungs may be removed by mucociliary transport. It also suggests that absorbed cerium is eliminated slowly from the body.

## Discussion

Progress in nanotechnology has produced a variety of nanoparticle generation systems which synthesize nanoparticles of desired size and properties. The in-house VENGES system employed in this study enabled us to control primary particle size and aerosol size distribution. This platform also allowed for in-flight coating of CeO_2_ with a nanothin layer of amorphous silica [21]. This flame-based silica-coating process has recently been explored as a means of high yield scalable manufacturing of silica-coated nanosized ENMs with cores of TiO_2_, Fe_2_O_3_, or Ag [36].

In this study, we sought to examine the effect of surface modification of CeO_2_ with amorphous silica on acute pulmonary responses as well as on CeO_2_ pharmacokinetics after IT instillation, gavage, and IV injection. We observed that exposure of rats to silica-coated CeO_2_ caused higher dose-dependent inflammatory responses compared to uncoated particles and a vehicle-only control group, as evidenced by increases in BAL parameters. However, the inflammatory effects induced by silica-coated CeO_2_ were transient and subsided by day 5 (Fig. 2d and e). This is consistent with our recent study in which 1 mg/kg dose of silica-coated CeO_2_ NPs also caused higher but transient inflammation [34]. We note that these findings are in contrast to our previously published report on the toxic and inflammatory effects of the same particles after inhalation exposure, where we showed that inhaled silica-coated CeO_2_ induced less toxicity and inflammation after exposure for 2 h per day for 4 consecutive days [28]. This discordance may be explained based on the higher doses used here and the different exposure method (bolus IT instillation vs. inspired aerosols over 8 h). Although IT instillation is a reliable method for administering a precise dose to the lungs, it differs from inhalation exposure in terms of particle distribution, dose rate, the extent of NP agglomeration and ressulting patterns of injury and clearance. Baisch et al. observed that inflammatory responses following intratracheal instillation were higher than those seen following whole body inhalation for single and repeated exposures of titanium dioxide NPs when deposited doses were comparable [37].

### Fate of intratracheally-instilled nanoceria

The lung clearance of uncoated CeO_2_ observed in this study was similar to our recent report on CeO_2_ NM-212. NM-212 was synthesized by a precipitation method unlike the CeO_2_ NPs used here which were flame-generated [38]. Our data are consistent with a study by He et al. where 63.9 ± 8.2 % of the intratracheally instilled dose still remained in the lungs after 28 days [31]. We found that the extent of silica-coated CeO_2_ clearance from the lung was significantly higher (~35 %) than uncoated CeO_2_ (~19 %). But an important finding was the significant influence of the silica coating on the lung clearance of CeO_2_ from days 2 to day 7. This period of more rapid clearance coincided with the initial phase characterized by greater inflammation and increased air-blood barrier permeability.

As the pulmonary surfactant lies at the outermost aspect of the air-blood barrier, inhaled and deposited NPs first encounter the biomolecules of the alveolar lining layer. This fluid consists of an ultra-thin layer of aqueous hypophase and a surface active lipoprotein mixture usually known as the pulmonary surfactant layer [39]. Pulmonary surfactant is composed of 85-90 % w/w phospholipids and 10 % w/w proteins [40]. Adsorption of phospholipids and proteins on the NP surface takes place rapidly [41]. Therefore, it is reasonable to assume that interactions of NPs with lung cells occur mostly with the NP-lipoprotein complex and not with bare NP surfaces [42]. Importantly, the adsorption of proteins and phospholipids on NPs may modulate their overall biological effects [43, 44].

We examined the protein corona formed on the surface of our test NPs as they encounter the lung lining fluid. The incubation of NPs in BAL fluid significantly increased their hydrodynamic sizes and changed the zeta potential of CeO_2_ NPs likely due to their interactions with phospholipids and proteins. Presumably, instilled NPs would immediately acquire protein coronas *in vivo* changing their surface charge and extent of aggregation unlike those in water suspension and in dry aerosols. The type of proteins comprising the corona may also impact NP translocation [45]. Aggregate size alterations could also influence the pulmonary effects and translocation of the core nanoceria. Notably, we found significantly more protein adsorbed in the “hard corona” of silica-coated compared to uncoated CeO_2_. The amounts of specific proteins comprising the hard corona shown in Fig. 5b were based on NP mass (μg/mg NPs). When expressed as amount of protein per unit surface area (μg/m^2^) of NPs, silica-coated CeO_2_ still bind more BAL proteins than uncoated NPs. Significantly more albumin, SP-A, α-1 antitrypsin, transferrin, and C3 proteins were present in the corona of silica-coated CeO_2_. These belong to the class of proteins that shuttle across the alveolar-epithelial barrier [46]. Receptor-mediated transport processes in the alveolar epithelium have been reported for albumin and transferrin [46]. Translocation of intratracheally instilled ^125^I-albumin from air spaces into the blood compartment has been reported previously [47]. Rapid translocation of synthetic organic NPs comprised of human serum albumin and a fluorophore has been demonstrated [48]. Whether this enhanced adsorption of albumin and transferrin onto silica-coated nanoceria contribute to their small but higher translocation through the lungs needs further investigation.

Studies have reported that some of the proteins present in BAL exhibit immunological functions (e.g., C3 and SP-A) [49–51]. It has been shown that coating of magnetite and TiO_2_ with SP-A improved their uptake in macrophages [52]. Our findings that the lipoprotein corona changes the agglomerate size and zeta potential of CeO_2_ also suggest that the corona can affect the manner in which alveolar macrophages interact, recognize, phagocytose, and process CeO_2_ NPs. Alveolar macrophages are the primary phagocytic cells for ultrafine particles in the lungs [53]. Particles may adhere to the surfaces of type I and type II epithelial cells as well, but lung parenchymal cells are less capable of phagocytosis [54]. AMs play a critical role in NP-induced inflammation and oxidative stress. Most of the deposited particles in the alveolar region are phagocytosed within a 24 h period after particle deposition, as long as the dose is not beyond the ingestion capacity of AMs [55, 56]. Notably, functionalized NPs are more effectively phagocytosed than non-functionalized NPs [57–60]. Recognition and phagocytosis of nanoparticles by AMs is a key component in nanoparticle dissolution and clearance.

We examined whether silica coating affects the distribution of CeO_2_ within the different lung compartments after the first 24 h post-instillation. We found no significant differences in the amount of radioactive CeO_2_ in lavaged alveolar cells, in cell-free supernatant, or in lavaged lungs. Furthermore, no significant difference was found in the number of AMs with internalized CeO_2_ NPs assessed by hyperspectral imaging of lavaged AMs. However, at 5 days post-instillation, significantly more AMs were found to have internalized silica-coated than uncoated CeO_2_. This enhanced uptake could be due to different corona profile, altered aggregate size or abundant recruitment of AMs observed with silica-coated CeO_2_. It is possible that this enhanced uptake of silica-coated CeO_2_ by activated AMs and the higher inflammation could lead to greater translocation of particles or particle-containing cells into the lymphatic system. For the lung parenchyma, clearance involves a slower phase, occurring in the alveoli. It consists of phagocytosis of particles from the lung surface by AMs and to a lesser extent by particles entering the lymphatics and subsequent accumulation in the regional lymph nodes.

We were unable to measure the lymphatic clearance of CeO_2_ NPs since lymph nodes were not included in this study. However, we have previously shown that ^65^Zn from ^65^ZnO NPs was more significantly translocated to tracheobronchial lymph nodes when coated similarly with amorphous silica [22]. Interestingly, despite the greater clearance from the lungs, ^141^Ce from silica-coated CeO_2_ was slightly lower in all the organs we examined (0.73 vs. 0.93 %). The cerium concentration retained in the liver, bone, kidneys, heart, and testes was lower. Excretion in the feces was also lower (12 vs. 19 %).

### Fate of ingested nanoceria

Data from animal and human studies show that inhaled nanoparticles are subject to different site-dependent clearance mechanisms [20]. These mechanisms include a fast clearance phase, which can be observed in the tracheobronchial region and is attributed to the mucociliary elimination with subsequent ingestion into the gastrointestinal tract and excretion via the feces. Thus, the oral exposure to nanoparticles is pertinent from an environmental exposure perspective, such as the ultrafine fraction of air pollution exposures. As a surrogate for entry of particles into the GI tract from the lungs, we also investigated the influence of silica coating on the bioavailability of CeO_2_ after gavage. Our data showed a rapid clearance of both types of CeO_2_. We found that nearly 100 % of the uncoated CeO_2_ and ~95 % of silica-coated CeO_2_ were eliminated in the feces within 7 days post-gavage. Despite the higher dose we used for gavage, there was negligible radioactivity in any organ or in urine samples collected over a period of 7 days. As has been demonstrated previously, neither CeO_2_ NP type cross the intestinal barrier nor is there dissolution followed by absorption [15, 32, 61].

### Fate of intravenously injected nanoceria

Due to increasing interest in CeO_2_ for potential nanomedical applications, we also investigated whether silica coating would affect the tissue distributions of IV-injected CeO_2_. Consistent with our earlier study [62], both CeO_2_ types were immediately taken up in organs rich in mononuclear phagocytes with direct access to the circulating blood, such as those in the liver (87 %), spleen (4 %), and bone (0.5 %). At 2 h, the total recovered ^141^Ce in all organs examined were 92.6 % (uncoated) and 92.2 % (silica-coated CeO_2_) of the total injected dose. Despite the significantly higher agglomerate size of uncoated nanoceria after interaction with plasma proteins, their liver uptake measured at 2 h was not different from silica-coated NPs. However, the silica coating enhanced the overall amount of cerium in some other organs. We found that binding of plasma proteins to the CeO_2_ surface was altered by the silica coating. Notably, bound albumin and α-2 hs glycoprotein were higher in silica-coated CeO_2_. A recent study showed that albumin-coated liposomes were taken up more efficiently than uncoated liposomes by murine macrophages [63]. The silica coating in our study also caused a significant reduction (6 %) in the liver retention of ^141^Ce with concomitant increases in the spleen and bone two days post-exposure. This likely reflects either enhanced dissolution of Kupffer cell-ingested silica-coated CeO_2_ or the release of intact NPs into the blood likely due to their smaller aggregate size (Table 1). Very small amounts of ^141^Ce (3.8-5.8 %) were cleared from the body two days post-exposure, indicating that absorbed cerium is biopersistent, as reported in other studies [32, 64].

## Conclusions

In summary, we found that silica coating of CeO_2_ caused a higher but transient lung inflammation and a higher lung clearance. It also altered the biodistribution of cerium when CeO_2_ were injected intravenously. These effects correlated with enhanced adsorption of proteins in lung lining fluid and plasma onto the silica coating. As surface chemistry greatly influences the formation of the nanoparticle corona, our future studies will focus on understanding nano-bio interactions with lung and plasma lipoproteins and their influence on toxicity and biokinetics of NPs.

## Methods

### Synthesis of CeO_2_ and silica-coated CeO_2_ nanoparticles

Detailed procedures of generating these nanoparticles have been reported [21, 28, 33]. Uncoated and SiO_2_-coated CeO_2_ nanoparticles were synthesized by flame spray pyrolysis (FSP) of cerium (III) ethylhexanoate (0.05 M) dissolved in xylene and cerium (III) ethylhexanoate (0.04 M) dissolved in xylene: EHA (3:1), respectively. The precursor solutions were fed through a stainless steel capillary at 5 ml/min, dispersed by 5 L/min O_2_ (Airgas, purity >99 %, pressure drop at nozzle tip: ρ_drop_ = 2 bar) and combusted to form the desired nanoparticles. A remixed stoichiometric methane-oxygen (1.5, 3.2 L/min) supporting flame was used in conjunction with 40 L/min O_2_ sheath gas. In the case of the synthesis of uncoated CeO_2_, 16 L/min of pure N_2_ was injected into the reactor through a torus ring with 16 equispaced and equisized (d_inner_ = 0.6 mm) jets at an injection height of 200 mm above the FSP burner. In the case of SiO_2_-coated CeO_2_, 16 L/min N_2_ carrying hexamethyldisiloxane (HMDSO, Sigma–Aldrich, St. Louis, MO, USA) vapor was fed through the same torus ring at an injection height of 300 mm. HMDSO vapor was obtained by bubbling 0.11 L/min gas through liquid HMDSO (300 ml) maintained at 11.3 °C using a temperature-controlled water bath. At saturation conditions, this corresponds to an HMDSO injection mass of 0.85 g/h into the reactor. In both cases, the reactor was enclosed above and below the torus ring by two quartz tubes (d_inner_ = 45 mm). Uncoated and silica-coated CeO_2_ NPs were collected on a water-cooled glass fiber filter (Whatman) located 80 cm above the reactor and stored in glass vials prior to experiments.

### Neutron activation of CeO_2_ nanoparticles

Both nanoparticle powders were neutron activated at the MIT Nuclear Reactor Laboratory (Cambridge, MA) with a thermal neutron flux of 5 x 10^13^ n/cm^2^/s for 24 h. The process generated the radioisotope ^141^Ce, which decays with a half-life of 32.5 days and emits gamma rays with an energy of 145.4 KeV. The specific activity was 2.7 μCi ^141^Ce per mg CeO_2_ and 3.4 μCi ^141^Ce per mg silica-coated CeO_2_.

### Animals

The protocols used in this study were approved by the Harvard Medical Area Animal Care and Use Committee. Male Wistar Han rats (8 weeks old) were obtained from Charles River Laboratories (Wilmington, MA) and were housed in standard microisolator cages under controlled conditions of temperature, humidity, and light at the Harvard Center for Comparative Medicine. They were fed commercial chow (PicoLab Rodent Diet 5053, Framingham, MA) and were provided with reverse-osmosis purified water *ad libitum*. The animals were acclimatized in the facility for at least 7 days before the start of experiments.

### Preparation of CeO_2_ nanoparticle suspensions for animal dosing

Particle suspensions at specified concentrations were prepared in sterile distilled water in conical polyethylene tubes. A critical dispersion sonication energy (DSE_cr_) to achieve the smallest particle agglomerate size was used, as previously reported [16]. The suspensions were sonicated at 242 J/ml (20 min/ml at 0.2 watt power output) in a cup sonicator fitted on Sonifier S-450A (Branson Ultrasonics, Danbury, CT, USA). The sample tubes were immersed in running cold water to minimize heating of the particles during sonication. The hydrodynamic diameter (D_H_), polydispersity index (PdI), and zeta potential (ζ) of each suspension were measured by dynamic light scattering using a Zetasizer Nano-ZS (Malvern Instruments, Worcestershire, UK).

### Assessment of pulmonary effects of CeO_2_ nanoparticles – Bronchoalveolar lavage and analyses

This experiment was performed to determine the influence of an amorphous silica coating on CeO_2_ pulmonary effects and also to identify a safe dose for pharmacokinetic studies on instilled materials. Thirty five rats (wt. = 267 ± 15 g) were instilled intratracheally with either uncoated or coated CeO_2_ NP suspensions at 0.2, 1.0, and 5 mg/kg (*n* = 5 rats/group). Another group of rats were instilled with an equivalent volume of distilled water and served as controls. The particle suspensions were delivered to the lungs through the trachea, as described earlier [35]. Twenty-four hours later, rats were anesthetized and then euthanized via exsanguination, with a cut in the abdominal aorta. The trachea was exposed and cannulated. The lungs were then lavaged 12 times with 3 mL of Ca^++^- and Mg^++^-free 0.9 % sterile PBS. The cells from all washes were separated from the supernatant by centrifugation (350 x g at 4 °C for 10 min). Total cell count and hemoglobin measurements were made from the cell pellets. A dilute cell suspension was cytocentrifuged, the cytospin was stained, and differential cell counting was performed. The supernatant from the first two washes was clarified via centrifugation (14,500 x g at 4 °C for 30 min), and used for standard spectrophotometric assays for LDH, MPO, and albumin [65].

### Pharmacokinetics of intratracheally-instilled, gavaged and intravenously injected ^141^CeO_2_ nanoparticles

The nanoparticle dose used for both NPs was 1 mg/kg for IT instillation, 1 mg/kg for IV injection, and 5 mg/kg for gavage administration. Neutron-activated ^141^CeO_2_ NPs were suspended in sterile distilled water at 0.67 mg/ml for IT instillation (1.5 ml/kg body weight) at 1 mg/ml for IV injection (1 ml/kg) or at 5 mg/ml for gavage administration (1 ml/kg) and sonicated as described above. The radioactivity in multiple aliquots of each suspension was measured in a WIZARD Gamma Counter (PerkinElmer, Inc., Waltham, MA).

Each rat was anesthetized with isoflurane (Piramal Healthcare, Bethlehem, PA). The ^141^CeO_2_ NP suspension was delivered to the lungs through the trachea, into the bloodstream via the penile vein, or into the stomach via the esophagus. Each rat was then placed in a metabolic cage with food and water *ad libitum* for fecal and urine sample collection. Five rats from the IT group were humanely sacrificed at 5 m, 2 d, 7 d and 28 d post-dosing. The same number of rats were analyzed at 5 m and 7 d post-gavage, and at 2 h and 2 d post-IV injection. Analysis of rats at 5 min post-IT instillation and post-gavage was performed to obtain an accurate measure of the initial deposited dose. Since we anticipated that clearance from the gastrointestinal tract would be relatively fast, the gavage experiment spanned only 7 days. Twenty four-hour samples of feces and urine were collected at selected time points (0–24 h, 2–3 days, 6–7 days, 9–10 days, 13–14 days, 20–21 days, and 27–28 days post-IT instillation; 0–24 h, 2–3 days, and 6–7 days post-gavage; and 0–24 h post-IV injection).

At each endpoint, rats were anesthetized and as much blood as possible was collected from the abdominal aorta. Plasma and red blood cells were separated by centrifugation at 3000 x g for 10 min at 4 °C. After euthanasia, the whole lungs, brain, heart, spleen, kidney, gastrointestinal tract, testes, liver, two femoral bones, and multiple samples of skeletal muscle, bone marrow, and skin were collected and placed in pre-weighed tubes. Each sample weight was recorded. Radioactivity was measured in a WIZARD Gamma Counter (PerkinElmer, Inc., Waltham, MA). Disintegrations per minute were calculated from the measured counts per minute (minus background) and the counter efficiency. Data were expressed as μCi/g and as a percentage of the administered dose retained in each organ. All radioactivity data were adjusted for physical decay over the entire observation period. The radioactivity in organs and tissues not measured in their entirety was estimated from measured aliquots as a percentage of total body weight as follows: skeletal muscle, 40 %; bone marrow, 3.2 %; peripheral blood, 7 %; skin, 19 %; and bone, 6 % [66, 67].

### Pulmonary distribution of ^141^CeO_2_ nanoparticles

To determine the pulmonary distribution of instilled ^141^CeO_2_ NPs within the lungs at 1 d post-instillation, a separate cohort of rats were IT-instilled with 1 mg/kg of either ^141^CeO_2_ or silica-coated ^141^CeO_2_. Twenty-four hours later, the lungs were lavaged as described above. The BAL fluid was centrifuged at 350 x g for 10 min at 4 °C to separate lavaged cells from the supernatant. The cell pellets were resuspended in 0.5 ml PBS. The lavaged lungs, BAL supernatants and cell pellets were analyzed for ^141^Ce. The total radioactivity in each of the three lung compartments was expressed as a percentage of the total radioactivity recovered in the whole lungs.

### Characterization of protein corona formation on CeO_2_ and silica-coated CeO_2_ nanoparticles in lung lining fluid and plasma

Nanoparticles (1 mg/mL) were incubated in 4 mL rat plasma for 30 min at 37 °C. Then, the suspension was centrifuged for 10 min at 14,500 x g. The resulting pellet was washed in DI water three times. After the final washing step, the NP pellet containing ‘hard corona’ was suspended in 20 μL of DI water to which 10 μL of 4x Laemmli sample buffer was added and vortexed. The sample was then heated to 95 °C for 7 min. After cooling to room temperature, 60 μL of mixed solution (57 μL Laemmli and 3 μL βME) was added to 18 μL of the sample. The samples were then loaded onto a gel and proteins were visualized by 1D SDS-PAGE in combination with Coomassie staining. Gel bands were excised and subjected to a modified in-gel trypsin digestion procedure [68]. Peptides were later extracted and then dried in a speed-vac (~1 h). The samples were then stored at 4 °C until analysis. On the day of analysis, the samples were reconstituted in 5–10 μL of HPLC solvent A (2.5 % acetonitrile, 0.1 % formic acid). A gradient was formed and peptides were eluted with increasing concentrations of solvent B (97.5 % acetonitrile, 0.1 % formic acid) [69]. Eluted peptides were subjected to electrospray ionization and then analyzed in an LTQ Orbitrap Velos Pro ion-trap mass spectrometer (Thermo Fisher Scientific, San Jose, CA). Peptides were detected, isolated, and fragmented to produce a tandem mass spectrum of specific fragment ions for each peptide. Peptide sequences (and protein identity) were determined by matching protein databases with the acquired fragmentation pattern by the software program, Sequest (ThermoFisher, San Jose, CA).

### Assessment of alveolar macrophage uptake of nanoceria in vivo

Non-radioactive CeO_2_ and silica-coated CeO_2_ NPs were instilled in a separate cohort of rats at the same dose and concentration (1 mg/kg, 0.67 mg/ml). At 1 or 5 days post-instillation, rats were sacrificed and their lungs lavaged as described above. BAL cells were cytocentrifuged and fixed on microscope slides. Uptake of nanoceria by cells was analyzed in an Olympus BX-41 microscope (CytoViva®, Auburn, AL) hyperspectral image analysis software. Each macrophage was scored for the presence of internalized NPs.

### Statistical analyses

Data were analyzed using multivariate analysis of variance (MANOVA) followed by Bonferroni (Dunn) *post hoc* tests using SAS Statistical Analysis Software (SAS Institute, Cary, NC). CytoViva data were analyzed by Student t test.
