# Erratum to: Silica coating influences the corona and biokinetics of cerium oxide nanoparticles

**DOI:** 10.1186/s12989-016-0146-4

**Published:** 2016-06-22

**Authors:** Nagarjun V. Konduru, Renato J. Jimenez, Archana Swami, Sherri Friend, Vincent Castranova, Philip Demokritou, Joseph D. Brain, Ramon M. Molina

**Affiliations:** 1 Molecular and Integrative Physiological Sciences Program, Department of Environmental Health, Harvard T.H. Chan School of Public Health, 665 Huntington Avenue, Boston, MA 02115 USA; 2 National Institute for Occupational Safety and Health, Morgantown, WV USA; 3 Department of Basic Pharmaceutical Sciences, School of Pharmacy, West Virginia University, P.O. Box 9530, Morgantown, WV 26506 USA

After the publication of this work [1] it was noticed that the values in Tables 4 and 5 were incorrect. The numbers should have been all multiplied by 10.

**Table 4:** Cerium concentration in different tissues at 2 hours after intravenous injection of uncoated or silica-coated CeO_2_ NPs

	CeO_2_	Silica-coated CeO_2_
	ng/g ± SE	ng/g ± SE
Lungs	143.7 ± 17.9	546.2 ± 49.3*
Liver	19517.9 ± 444.9	16636.8 ± 676.7*
Bone	33.8 ± 4.4	64.2 ± 11.4*
Cecum	6.2 ± 4.3	2.1 ± 0.3
Large intestine	3.6 ± 0.9	2.8 ± 0.5
Bone marrow	17.3 ± 3.4	467 ± 7.0*
Spleen	16807.1 ± 3579.1	12043.8 ± 1717.6
Stomach	37.7 ± 16.7	47.7 ± 23.8
Kidneys	30.9 ± 14.4	52.7 ± 2.9
Small intestine	13.7 ± 2.0	11.2 ± 1.4
Heart	7.7 ± 1.5	14.1 ± 2.6
Testes	0.9 ± 0.2	1.9 ± 0.1*
Skeletal muscle	1.6 ± 0.5	2.3 ± 0.1
Brain	0.4 ± 0.0	1.1 ± 0.2*
Skin	2.0 ± 0.3	6.8 ± 0.4*
Plasma	1.4 ± 0.3	1.4 ± 0.3
RBC	26.6 ± 5.1	20.7 ± 2.3

Data are mean ± SE ng/g cerium concentration, *n =* 5/group

Ce concentration was estimated (ng/μCi_NPs_ x μCi/g_tissue_)

*P < 0.05, CeO_2_ vs. silica-coated CeO_2_

**Table 5:** Cerium concentration in different tissues at 2 days after intravenous injection of uncoated or silica-coated CeO_2_ NPs

	CeO_2_	Silica-coated CeO_2_
	ng/g ± SE	ng/g ± SE
Lungs	56.5 ± 15.3	231.5 ± 28.3*
Liver	19044.3 ± 932.2	16525.7 ± 380.7*
Bone	27.2 ± 6.4	109.5 ± 16.1*
Cecum	1.5 ± 0.2	9.5 ± 2.6*
Large intestine	2.1 ± 0.3	10.7 ± 2.9*
Bone marrow	14.2 ± 2.8	66.4 ± 12.3*
Spleen	10960.2 ± 3334.9	18223.3 ± 1818.3
Stomach	20.1 ± 8.9	32.9 ± 12.5
Kidneys	13.2 ± 1.8	63.2 ± 5.8*
Small intestine	14.7 ± 1.4	6.3 ± 1.2*
Heart	7.5 ± 2.6	10.5 ± 1.2
Testes	0.2 ± 0.1	2.0 ± 0.2*
Skeletal muscle	0.5 ± 0.1	1.8 ± 0.2*
Brain	0.2 ± 0.1	0.8 ± 0.2*
Skin	1.9 ± 0.3	5.2 ± 0.8*
Plasma	0.2 ± 0.1	0.9 ± 0.2*
RBC	3.0 ± 1.0	6.9 ± 0.3*

Data are mean ± SE ng/g cerium concentration, *n =* 5/group

Ce concentration was estimated (ng/μCi_NPs_ x μCi/g_tissue_)

*P < 0.05, CeO_2_ vs. silica-coated CeO_2_

Please see below corrected tables 4 and 5.
